# Supplementary material for: The association between local hospital segregation and hospital quality for medicare enrollees
Source: PLoS One. 2025 Dec 5;20(12):e0337559. doi: 10.1371/journal.pone.0337559 (PMC12680329; doi:10.1371/journal.pone.0337559)

**Supporting Information: The Association Between Local Hospital Segregation and Hospital Quality for Medicare Enrollees**

**Table of Contents**

**A.2 Sample composition**

**A.2 Sample composition**

In S1 Figure , we outline how we constructed our dataset.

**S1 Figure. Sample construction**


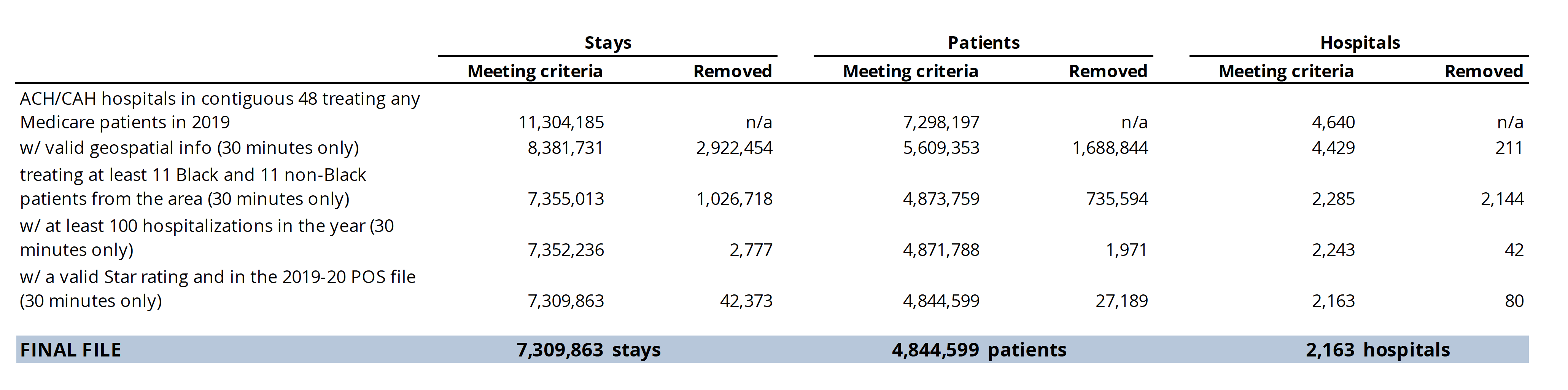

Supplement: S1 Fig — (DOCX) [file pone.0337559.s001.docx]
