# Supplementary material for: The association between local hospital segregation and hospital quality for medicare enrollees
Source: PLoS One. 2025 Dec 5;20(12):e0337559. doi: 10.1371/journal.pone.0337559 (PMC12680329; doi:10.1371/journal.pone.0337559)
Supplement: S2 Fig — (DOCX) [file pone.0337559.s002.docx]

**Supporting Information: The Association Between Local Hospital Segregation and Hospital Quality for Medicare Enrollees**

**Table of Contents**

**A.3 Logistics and ordered logistics regression results**

**S2. Figure. Hospital Star Standardized Group Scores**

Panel A: Model 1


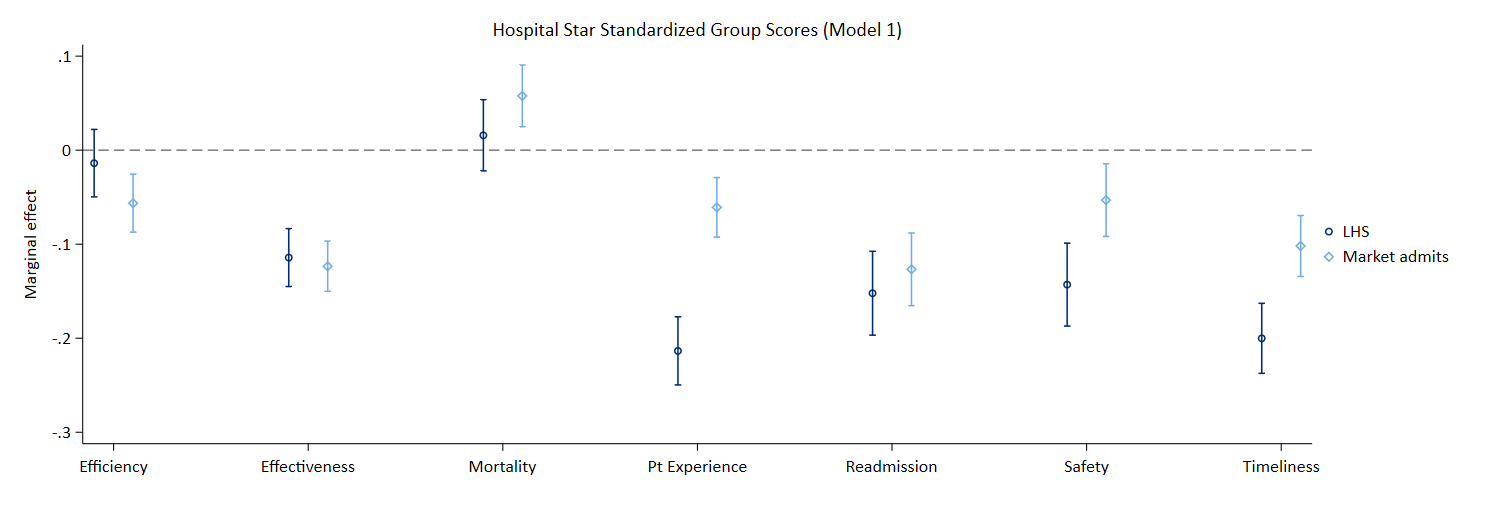


Panel B: Model 2


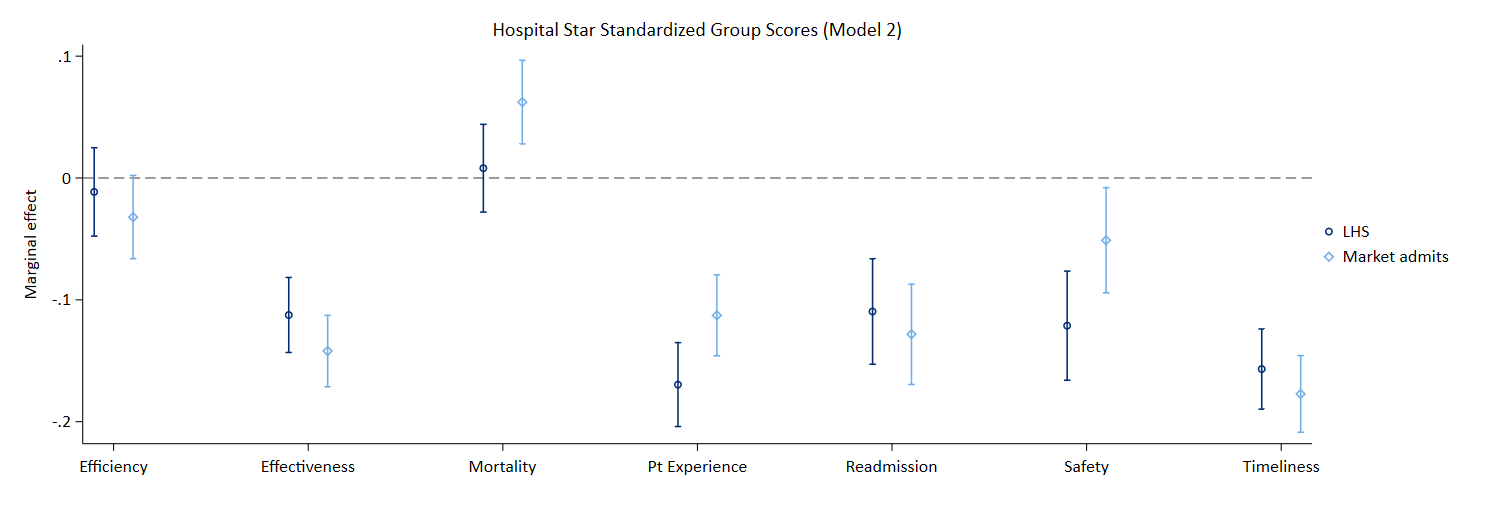


Note: *Market admits = the percentage of hospital admissions in a hospital's market area of Black patients.
