# Supplementary material for: The association between local hospital segregation and hospital quality for medicare enrollees
Source: PLoS One. 2025 Dec 5;20(12):e0337559. doi: 10.1371/journal.pone.0337559 (PMC12680329; doi:10.1371/journal.pone.0337559)
Supplement: S1 Table — (DOCX) [file pone.0337559.s003.docx]

**Supporting Information: The Association Between Local Hospital Segregation and Hospital Quality for Medicare Enrollees**

**Table of Contents**

**A.1 CMS Star Quality Score components definitions and examples**

| A.1. CMS Star Quality Score components definitions and examples  This is reproduced from CMS information, with sources cited below.  S1 Table : CMS Overall Hospital Quality Star Rating (2020) group score definitions | | | | |
| --- | --- | --- | --- | --- |
| Group score domain | **Definition** | **Example** | **Weight** |  |
| Mortality | Deaths within 30 days of a hospital admission from any cause related to medical conditions, including heart attack (AMI), heart failure (HF), pneumonia (PN), chronic obstructive pulmonary disease (COPD), and stroke; as well as surgical procedures, including coronary artery bypass graft (CABG). (7 measures) | Death rate for heart attack patients | 22% |  |
| Safety of care | Six Healthcare-Associated Infections (HAI) measures; rate of complications for hip/knee replacement patients; and one composite measure of serious complications (CMS Patient Safety Indicator [PSI]-90). (8 measures) | Central line-associated bloodstream infections (CLABSI) in ICUs and select wards | 22% |  |
| Readmission | Unplanned readmission to any acute care hospital within 30 days of discharge from a hospitalization for any cause related to medical conditions, including AMI, HF, PN and COPD; and surgical procedures, including hip/knee replacement and CABG, and colonoscopy procedures. (9 measures) | Hospital return days for pneumonia patients | 22% |  |
| Patient experience | Selected responses from the Hospital Consumer Assessment of Healthcare Providers and Systems (HCAHPS) (10 measures) | Patients who reported that their doctors communicated well | 22% |  |
| Effectiveness of care | Percentage of hospital patients who got treatments known to get the best results for certain common, serious medical conditions or surgical procedures; how quickly hospitals treat patients who come to the hospital with certain medical emergencies; and how well hospitals provide preventive services. These measures only apply to patients for whom the recommended treatment would be appropriate. (10 measures) | Healthcare personnel vaccination | 4% |  |
| Timeliness of care | See above, **Effectiveness of care**. (8 measures) | Percentage of patients who left the emergency department before being seen | 4% |  |
| Efficient use of medical imaging | The purpose of reporting these measures is to reduce unnecessary exposure to contrast materials and/or radiation, to ensure adherence to evidence-based medicine and practice guidelines, and to prevent wasteful use of Medicare resources. The measures only apply to Medicare patients treated in hospital outpatient departments. (5 measures) | Colonoscopy interval for patients with a history of adenomatous polyps – avoidance of inappropriate use | 4% |  |
| Note: The Overall Hospital Quality Star Rating is based on adequate information regarding hospital quality. CMS evaluated and developed standards regarding the minimum number of measures and groups a hospital must report to receive a publicly reported Overall Star Rating on Care Compare. CMS set these thresholds to allow for as many hospitals as possible to receive an Overall Star Rating without sacrificing the validity and reliability of the Overall Star Rating methodology #. Note that these percentage weights are out of 100%. If a hospital has no measures in a certain measure group, the weighted percentage is redistributed proportionally to the other measure groups.  Sources:   - <https://data.cms.gov/provider-data/topics/hospitals/about-data#measure-update-frequency> - Measure counts: Overall Hospital Quality Star Rating on Hospital Compare: Comprehensive Methodology Report (v3.0) ([link](https://qualitynet.cms.gov/inpatient/public-reporting/overall-ratings/resources)) - Specific measures: January 2020 Overall Star Rating HSR User Guide (HUG) ([link](https://qualitynet.cms.gov/inpatient/public-reporting/overall-ratings/reports)) | | | | |
