# Supplementary material for: The association between local hospital segregation and hospital quality for medicare enrollees
Source: PLoS One. 2025 Dec 5;20(12):e0337559. doi: 10.1371/journal.pone.0337559 (PMC12680329; doi:10.1371/journal.pone.0337559)
Supplement: S2 Table — (DOCX) [file pone.0337559.s004.docx]

**Supporting Information: The Association Between Local Hospital Segregation and Hospital Quality for Medicare Enrollees**

**Table of Contents**

**A.3 Logistics and ordered logistics regression results**

**Appendix A.3: Additional Logistics Regressions**

**S2 Table. Association between hospital quality (Star rating) and hospital characteristics using CMS data from 2019: Ordered logit results**

|  | **1-Star** | **2-Star** | **3-Star** | **4-Star** | **5-Star** |
| --- | --- | --- | --- | --- | --- |
|  |  |  |  |  |  |
| LHS (10 pctg pts) | 0.026  (.020,.033) | 0.040  (.031,.050) | <0.001  (-.003,.003) | -0.037  (-.046,-.028) | -0.030  (-.038,-.022) |
|  |  |  |  |  |  |
| Market admits* (10 pctg pts) | 0.019  (.013,.026) | 0.030  (.021,.039) | <0.001  (-.002,.002) | -0.027  (-.036,-.019) | -0.022  (-.029,-.015) |
|  |  |  |  |  |  |
| Market size |  |  |  |  |  |
| <2 (ref) |  |  |  |  |  |
| 3 to 6 | 0.004  (-.015,.022) | 0.005  (-.021,.031) | <0.001  (-.003,.002) | -0.005  (-.029,.020) | -0.003  (-.021,.0147) |
| 7 or more | -0.018  (-.036,.0003) | -0.028  (-.055,-.001) | -0.001  (-.003,.002) | 0.025  (.0004,.051) | 0.021  (.001,.041) |
|  |  |  |  |  |  |
| Ownership |  |  |  |  |  |
| Private/church (nonprofit) (ref) |  |  |  |  |  |
| Physician/other | <0.001  (-.018,.018) | <0.001  (-.034,.035) | <0.001  (-.006,.006) | <0.001  (-.031,.030) | <0.001  (-.028,.027) |
| Private (for profit) | 0.063  (.042,.085) | 0.09  (.064,.115) | -0.011  (-.021,-.002) | -0.084  (-.109,-.060) | -0.058  (-.073,-.042) |
| Government | 0.038  (.017,.059) | 0.061  (.031,.090) | -0.001  (-.008,.005) | -0.056  (-.084,-.028) | -0.042  (-.060,-.023) |
|  |  |  |  |  |  |
| Teaching hospital=1 | 0.033  (.020,.046) | 0.053  (.032,.074) | <0.001  (-.003,.004) | -0.049  (-.068,-.030) | -0.038  (-.052,-.023) |
|  |  |  |  |  |  |
| DSH status=1 | 0.057  (.045,.069) | 0.116  (.088,.144) | 0.038  (.019,.057) | -0.101  (-.124,-.078) | -0.11  (-.146,-.075) |
|  |  |  |  |  |  |
| Region |  |  |  |  |  |
| Northeast (ref) |  |  |  |  |  |
| Midwest | -0.096  (-.124,-.068) | -0.128  (-.159,-.096) | 0.018  (.002,.034) | 0.121  (.091,.151) | 0.085  (.062,.108) |
| South | -0.077  (-.106,-.048) | -0.092  (-.120,-.064) | 0.025  (.010,.039) | 0.089  (.061,.117) | 0.055  (.038,.072) |
| West | -0.07  (-.100,-.040) | -0.081  (-.114,-.047) | 0.025  (.011,.040) | 0.078  (.047,.110) | 0.047  (.027,.068) |

**Note**. Values in the table are marginal effects with confidence intervals in parentheses. LHS=Local Hospital Segregation index; DSH=disproportionate share hospital. *Market admits = the percentage of hospital admissions in a hospital’s market area among Black patients.
