# Supplementary material for: The association between local hospital segregation and hospital quality for medicare enrollees
Source: PLoS One. 2025 Dec 5;20(12):e0337559. doi: 10.1371/journal.pone.0337559 (PMC12680329; doi:10.1371/journal.pone.0337559)
Supplement: S3 Table — (DOCX) [file pone.0337559.s005.docx]

**Supporting Information: The Association Between Local Hospital Segregation and Hospital Quality for Medicare Enrollees**

**Table of Contents**

**A.3 Logistics and ordered logistics regression results**

**S3 Table. Association between the probability of being admitted to a low-quality hospital (1- or 2-Star)**

**and the Local Hospital Segregation Index: Logistics results**

| **LHS Quintile** | **Average LHS**  **(10 percentage pts)** | **Model 1** | **Model 2** |
| --- | --- | --- | --- |
| Quintile 1 | -1.091 (0.531) | (ref) | (ref) |
| Quintile 2 | -0.271 (0.112) | 0.093 (0.034,0.153) | 0.082 (0.021,0.142) |
| Quintile 3 | -0.025 (0.038) | 0.140 (0.077,0.204) | 0.117 (0.052,0.182) |
| Quintile 4 | 0.159 (0.093) | 0.197 (0.135,0.259) | 0.180 (0.116, 0.244) |
| Quintile 5 | 1.443 (1.275) | 0.232 (0.171,0.291) | 0.178 (0.119, 0.238) |

**Note.** Values in the right two columns are marginal effects with confidence intervals in parentheses. Model 1 = adjusted for LHS and % Black residents from the market admitted to any hospital. Model 2 = additionally adjusted for market size, ownership, teaching status, disproportionate share hospital (DSH) status, and Census region. LHS=Local Hospital Segregation index.
