# Supplementary material for: The association between local hospital segregation and hospital quality for medicare enrollees
Source: PLoS One. 2025 Dec 5;20(12):e0337559. doi: 10.1371/journal.pone.0337559 (PMC12680329; doi:10.1371/journal.pone.0337559)
Supplement: S4 Table — (DOCX) [file pone.0337559.s006.docx]

**Supporting Information: The Association Between Local Hospital Segregation and Hospital Quality for Medicare Enrollees**

**S4 Table. Association between hospital quality and hospital characteristics: Logistics (1- or 2-Star) and OLS (group scores) results**

|  | - 1. **Star Rating** | **Group Score: Efficiency** | **Group Score: Effectiveness** | **Group Score: Mortality** | **Group Score: Patient Experience** | **Group Score: Readmission** | **Group Score: Safety** | **Group Score: Timeliness** |
| --- | --- | --- | --- | --- | --- | --- | --- | --- |
|  |  |  |  |  |  |  |  |  |
| LHS (10 pctg pts) | 0.06 | -0.011 | -0.112 | 0.008 | -0.17 | -0.11 | -0.121 | -0.157 |
|  | (.041,.079) | (-.048,.025) | (-.143,-.082) | (-.028,.044) | (-.204,-.135) | (-.153,-.066) | (-.166,-.076) | (-.190,-.124) |
| Market admits* (10 pctg pts) | 0.041 | -0.032 | -0.142 | 0.062 | -0.113 | -0.128 | -0.051 | -0.177 |
|  | (.023,.058) | (-.066,.002) | (-.171,-.113) | (.028,.097) | (-.146,-.080) | (-.169,-.087) | (-.094,-.008) | (-.209,-.146) |
| Market size |  |  |  |  |  |  |  |  |
| <2 (ref) |  |  |  |  |  |  |  |  |
| 3 to 6 | 0.012 | -0.038 | 0.032 | 0.044 | -0.023 | -0.046 | -0.034 | -0.209 |
|  | (-.043,.067) | (-.142,.065) | (-.056,.121) | (-.059,.148) | (-.123,.078) | (-.170,.079) | (-.164,.097) | (-.304,-.114) |
| 7 or more | -0.015 | 0.076 | 0.142 | 0.542 | -0.01 | -0.138 | -0.148 | -0.294 |
|  | (-.071,.041) | (-.031,.182) | (.050,.233) | (.436,.649) | (-.112,.093) | (-.266,-.010) | (-.281,-.014) | (-.391,-.196) |
|  |  |  |  |  |  |  |  |  |
| Ownership |  |  |  |  |  |  |  |  |
| Private/church (nonprofit) (ref) |  |  |  |  |  |  |  |  |
| Physician/other | 0.031 | -0.039 | 0.137 | 0.003 | 0.046 | 0.048 | -0.029 | 0.038 |
|  | (-.037,.098) | (-.167,.088) | (.030,.245) | (-.124,.131) | (-.073,.165) | (-.103,.199) | (-.186,.127) | (-.077,.153) |
| Private (for profit) | 0.161 | 0.006 | 0.304 | 0.003 | -0.515 | -0.546 | 0.179 | 0.394 |
|  | (.108,.214) | (-.092,.103) | (.221,.387) | (-.094,.101) | (-.607,-.423) | (-.663,-.429) | (.059,.300) | (.304,.483) |
| Government | 0.066 | -0.154 | -0.198 | -0.099 | -0.061 | -0.195 | -0.172 | -0.046 |
|  | (.008,.125) | (-.263,-.044) | (-.292,-.104) | (-.209,.010) | (-.167,.045) | (-.327,-.064) | (-.310,-.034) | (-.147,.054) |
|  |  |  |  |  |  |  |  |  |
| Teaching hospital=1 | 0.094 | 0.047 | 0.039 | -0.011 | -0.198 | -0.189 | -0.041 | -0.313 |
|  | (.054,.135) | (-.029,.123) | (-.026,.104) | (-.087,.065) | (-.269,-.127) | (-.280,-.098) | (-.135,.053) | (-.382,-.244) |
|  |  |  |  |  |  |  |  |  |
| DSH status=1 | 0.197 | -0.185 | -0.075 | -0.235 | -0.431 | -0.299 | -0.192 | -0.106 |
|  | (.144,.250) | (-.304,-.067) | (-.174,.023) | (-.352,-.118) | (-.546,-.317) | (-.437,-.161) | (-.342,-.042) | (-.212,.001) |
|  |  |  |  |  |  |  |  |  |
| Region |  |  |  |  |  |  |  |  |
| Northeast (ref) |  |  |  |  |  |  |  |  |
| Midwest | -0.223 | 0.051 | 0.155 | -0.158 | 0.417 | 0.598 | 0.349 | 0.9 |
|  | (-.289,-.157) | (-.072,.174) | (.050,.261) | (-.281,-.034) | (.301,.533) | (.450,.747) | (.196,.502) | (.787,1.013) |
| South | -0.141 | -0.18 | 0.102 | -0.441 | 0.407 | 0.603 | 0.264 | 0.754 |
|  | (-.205,-.077) | (-.295,-.064) | (.003,.201) | (-.557,-.325) | (.299,.515) | (.464,.742) | (.121,.407) | (.648,.860) |
| West | -0.138 | 0.097 | -0.086 | -0.237 | 0.061 | 0.842 | 0.256 | 0.121 |
|  | (-.209,-.067) | (-.036,.230) | (-.197,.025) | (-.368,-.106) | (-.060,.183) | (.686,.998) | (.095,.417) | (.002,.240) |

**Note**. Values in the table are marginal effects with confidence intervals in parentheses. LHS=Local Hospital Segregation index; DSH=disproportionate share hospital. *Market admits = the percentage of hospital admissions in a hospital’s market area among Black patients.
