# Supplementary material for: The association between local hospital segregation and hospital quality for medicare enrollees
Source: PLoS One. 2025 Dec 5;20(12):e0337559. doi: 10.1371/journal.pone.0337559 (PMC12680329; doi:10.1371/journal.pone.0337559)
Supplement: S5 Table — (DOCX) [file pone.0337559.s007.docx]

**Supporting Information: The Association Between Local Hospital Segregation and Hospital Quality for Medicare Enrollees**

**Table of Contents**

**A.4 Logistics results using a 15-minute driving radius**

**S5 Table. Association between hospital quality (1- or 2-Star rating) and hospital characteristics: Market definition using a 15-minute driving radius**

|  | **Model 1** | **Model 2** | **Reference 30-Minute Radius (Model 2)*** |
| --- | --- | --- | --- |
| LHS (10 pctg pts) | 0.079  (.048, .109) | 0.058  (.028,.088) | 0.060  (.041,.079) |
|  |  |  |  |
| Market admits** (10 pctg pts) | 0.038  (.025, .052) | 0.042  (.028,.056) | 0.041  (.023,.058) |
|  |  |  |  |
| Market size |  |  |  |
| <2 (ref) |  |  |  |
| 3 to 6 |  | -0.021  (-.068, .026) | 0.012  (-.043,.067) |
| 7 or more |  | -0.019  (-.088,.050) | -0.015  (-.071,.041) |
| Ownership |  |  |  |
| Private/church (nonprofit) (ref) |  |  |  |
| Physician/other |  | 0.036  (-.037,.109) | 0.031  (-.037,.098) |
| Private (for profit) |  | 0.177  (.121, .233) | 0.161  (.108,.214) |
| Government |  | 0.075  (.013,.137) | 0.066  (.008,.125) |
|  |  |  |  |
| Teaching hospital=1 |  | 0.09  (.048,.134) | 0.094  (.054,.135) |
|  |  |  |  |
| DSH status=1 |  | 0.184  (.123, .244) | 0.197  (.144,.250) |
|  |  |  |  |
| Region |  |  |  |
| Northeast (ref) |  |  |  |
| Midwest |  | -0.250  (-.321,-.179) | -0.223  (-.289,-.157) |
| South |  | -0.162  (-.227,-.096) | -0.141  (-.205,-.077) |
| West |  | -0.151  (-.228,-.073) | -0.138  (-.209,-.067) |

**Note**. Values in the table are marginal effects with confidence intervals in parentheses. We did not calculate an LHS for any hospital in the main cohort that had too few eligible patients in its 15-minute radius (N=1,961 hospitals in the minimally adjusted model / 1,956 hospitals in the fully adjusted model presented in this table). LHS=Local Hospital Segregation index; DSH=disproportionate share hospital. *Reference coefficient estimates for 30-minute radius, reproduced from Table A.3.a above. **Market admits = the percentage of hospital admissions in a hospital’s market area among Black patients.
