# Supplementary material for: The association between local hospital segregation and hospital quality for medicare enrollees
Source: PLoS One. 2025 Dec 5;20(12):e0337559. doi: 10.1371/journal.pone.0337559 (PMC12680329; doi:10.1371/journal.pone.0337559)
Supplement: Attachment — Submitted filename: Response to Reviewers.docx [file pone.0337559.s008.docx]

Dear Dr. Ko:

Thank you for the opportunity to resubmit our paper “The Association Between Local Hospital Segregation and Hospital Quality for Medicare Enrollees” (PONE-D-25-24400) for consideration by PLOS One. Your suggestions along with those of the referees were very helpful in preparing the resubmission. Below we consider each of the suggestions in turn:

1. *The referees have offered a few suggestions to potentially assuage their concerns. On my read, I have to say that you need to add more details on the paper's contribution to literature (Referee 1). Also, I agree with Referee 2 that your paper would benefit from discussing more on mechanisms. Because of the importance of the question, I am willing to give you another round to try. I like the question you pursue and am willing to hear if you have a response to the concerns and thoughts raised by the referees.*

Thank you again for the opportunity to resubmit. We have provided stronger discussions of both the paper’s contribution to the literature and potential mechanisms; these are detailed below.

**Reviewer #1**:

2. *My biggest concern is that the observed associations may be driven by markets with a small number of hospitals. LHS values are more likely to be extreme due to the “law of small numbers” where extreme outcomes are more common in small samples. In this study, roughly 20 percent of markets have only one or two hospitals, and about 50 percent have six or fewer. Since LHS values are likely to be more extreme in these small markets, the associations with hospital quality may be disproportionately driven by these cases. It would be valuable to repeat the analysis restricting to markets with at least 7 or 10 hospitals to assess the robustness of the findings in settings less susceptible to this small-sample variance artifact.*

This is an excellent suggestion, so we reran our models stratified by the number of hospitals in each market, and presented the coefficients in Exhibit 3. In the methods section, we described our approach:

First, we hypothesize that in smaller markets with the highest concentration – for example those with just 2 hospitals –we would observe the greatest degree of sorting and adverse effects on hospital quality; thus we stratified our analysis by the number of hospitals in the market: small (1-2 hospitals), medium (3-6 hospitals), and large (7+ hospitals).

In the results section, we further discuss the results from Exhibit 3:

The next three rows presents results for Model 1 and Model 2, stratified by the size of the market. For the small market, with 1-2 hospitals, the coefficient in Model 1 on LHS is 0.046, and not significantly different from zero; in medium-sized markets with 3-6 hospitals, the coefficient is 0.089 (95% CI, 0.031-0.147), and for the large-sized markets (7+) the coefficient is 0.076 (95% CI, 0.055-0.096); the pattern of coefficients is similar, but attenuated for Model 2. That is, the association between LHS and our quality measures are the strongest among the large and especially medium-sized markets, suggesting a greater degree of segmentation within larger markets.

3. *The results are consistent with structural racial disparities in healthcare delivery and hospital quality, but the interpretation appears to lean toward a supply-side explanation. Implicitly, the findings assume that both Black and non-black patients both have full information about hospital quality. However, it may be the case that Black patients face greater information gaps regarding hospital quality, while non-Black patients have better access to such information. This alone could lead to differential sorting, even in the absence of discrimination or exclusion. In the presence of such information gaps, patients may rely on heuristics such as choosing the same hospitals as others in their community. This could reinforce patterns of segregation and sorting. Such behavior would still be consistent with the observed results but would reflect limited information or social learning rather than active exclusion. It would strengthen the paper to include a discussion of these potential demand-side mechanisms and to acknowledge that observed sorting may partly reflect differences in information, trust, or social networks.*

We can concede that information gaps on hospital quality may play a role in patterns of hospital choice but there is evidence in the literature that demonstrates that supply-side mechanism play a significantly larger role. In the discussion section, we include this paragraph to address these concerns:

Our findings demonstrate that while information gaps regarding hospital quality may contribute to observed patterns of hospital choice, they are insufficient to fully explain the persistent sorting of Black patients into lower-quality hospitals. Our analyses, focused on Medicare fee-for-service beneficiaries who have broad nominal choice among hospitals, show that disparities persist even when several hospitals are located within close geographic proximity and quality information is publicly available(9). This is consistent with prior research showing that, although all patients—including Black patients—use social networks and trust in their communities during decision-making, these behaviors alone do not account for the magnitude of observed segregation in hospital use (7, 8). Rather, the literature and our own results indicate that structural, economic, and institutional factors—such as insurance networks, hospital referral patterns, historical relationships, and experiences of inclusion or exclusion within health care settings—play a predominant role in shaping hospital selection for Black patients(8, 9, 29). Our results, particularly for emergency care where patient choice is less likely to be important, reinforce the conclusion that supply-side constraints and systemic barriers, rather than information (and demand more generally), underpin the persistent inequities in hospital quality experienced by Black communities(29, 30).

3. *The analytic sample drops from over 4,400 to 2,285 hospitals due to the exclusion of hospitals with fewer than 11 Black or 11 non-Black admissions. Given the size of this reduction, it would be helpful for the authors to report how many hospitals were excluded for each criterion separately (i.e., low Black vs. low non-Black counts). Please also*

Thank you for this suggestion. We now describe the sample in more detail, including as well data on the number of hospital admissions excluded. Because the roughly half of our sample comprises really small hospitals (or even rehabilitation hospitals), we only lose 12.8% of admissions from our original sample of “within-market” admissions. Our updated text is as follows:

We identified 4,640 acute care or critical access hospitals providing treatment to any 2019 Medicare fee-for-service beneficiaries across 48 states (for a total of 11,304,184 admissions); our initial sample is the 8,381,731 hospital admissions with valid geospatial information within 30 minutes of the admitting hospital. We excluded 211 hospitals lacking the necessary geospatial data to construct the market area, resulting in 4,429 hospitals. In compliance with CMS reporting requirements, we dropped hospitals with fewer than 11 Black patients with eligible hospitalizations (which led to dropping 2,063 hospitals), fewer than 11 patients not identifying as Black (5 hospitals), and with fewer than 11 patients for both groups (76 hospitals), leading to 2,285 hospitals. For statistical precision, we removed 42 additional hospitals with fewer than 100 total hospitalizations of area Medicare patients aged 65 years or older, resulting in 2,243 hospitals. As a final step, we excluded 80 hospitals without a 2020 Star Rating. Thus, our exclusion rules resulted in slightly less than half of the original count, 2,163 hospitals, with 12.8% fewer admissions (7,309,863 in total).

4. *It may also be useful to examine whether the associations between LHS and hospital quality are strongest in markets where the index hospital is the only one serving a meaningful number of Black patients. This could help clarify whether the observed quality disparities are concentrated in racially homogenous markets where all other hospitals are nearly “all-White.”*

This is a great suggestion. To test this hypothesis, we allowed for a nonlinear effect of the exposure on the quality of the hospital by creating quintiles for the LHS; thus the fifth quintile includes those hospitals with the highest LHS (and thus would correspond to the single hospital in a market serving largely Black patients). In the methods section we describe this (and the other sensitivity analysis) as follows:

Second, we hypothesize that hospitals with the highest LHS experience the sharpest increase in the probability of being lower-quality (one or two star) hospitals; we do this by stratifying hospitals into quintiles depending on their LHS.

And in the results section:

We also present results stratified by the degree of concentration in the market. The next three rows present results for Model 1 and Model 2, stratified by the size of the market. For the small market, with 1-2 hospitals, the coefficient in Model 1 on LHS is 0.046, and not significantly different from zero; in medium-sized markets with 3-6 hospitals, the coefficient is 0.089 (95% CI, 0.031-0.147), and for the large-sized markets (7+) the coefficient is 0.076 (95% CI, 0.055-0.096); the pattern of coefficients is similar, but attenuated for Model 2. That is, the association between LHS and our quality measures are the strongest among the large and especially medium-sized markets, suggesting a greater degree of segmentation within markets (see Exhibit 3).

And later:

In Appendix Table A.3.b, we provide the estimates of both Model 1 and Model 2 but with hospitals stratified into quintiles where the lowest quintile exhibits a negative LHS and the highest quintile measures hospitals with the highest LHS. Surprisingly, we find that the largest jump in the probability of being admitted to a 1- or 2-star hospital arises in moving from Quintile 1 (the reference) to Quintile 2, with little change in the probability arising from a shift from Quintile 4 to Quintile 5. That is, the hospitals in locally segregated markets with a large (in magnitude) negative LHS exhibit the lowest likelihood of being 1- or 2-star hospitals.

5*. Other clarifications:
o Clarify whether these excluded hospitals are included in the denominator when calculating the market-level Black admission share for the LHS index. I could not see it in the manuscript.*

An excellent point. We have clarified this in the manuscript in the methods section with the following text:

Hospitals excluded from the analytic sample were not included in the denominator when estimating the market-level Black admission share for the LHS index. The actual share of Black admissions in a given hospital is equal to the LHS plus the market-level fraction of Black admissions; thus, it is a decomposition by which we can identify separately the association between hospital star ratings and (a) the market-level degree of segregation and (b) the within-market segregation as measured by the LHS.

*o The sentence stating that “a hospital value of -0.10… is 10% less than that in the market area” is misleading. Since the LHS measures an absolute difference, it should read “10 percentage points lower” to reflect the correct interpretation.*

Correct, thank you for the clarification; this has been corrected.

*o In the regression models, the market-level fraction of Black admissions is included as a control alongside the LHS index. Please clarify whether this variable is calculated using the same method as in the LHS denominator, specifically whether the index hospital is excluded from this calculation.*

Yes, this is calculated using the same method as the LHS denominator; we have clarified this in the text:

The market-level fraction of Black admissions is common to all hospitals in that market.

As discussed in our earlier paper, the actual share of Black admissions in a given hospital is equal to the LHS plus the market-level fraction of Black admissions; thus, it is a decomposition by which we can identify separately the association between hospital star ratings and (a) the market-level degree of segregation and (b) the within-market segregation as measured by the LHS.

**Reviewer #2**

6. *This paper examines the association between Local Hospital Segregation (LHS) and Hospital Quality. Using 2020 hospital star ratings and 2019 patient flow from Medicare Claims to construct the LHS measure, the authors provide cross-sectional evidence that hospitals with a higher local hospital segregation index (i.e., admitting disproportionally more black patients) tend to be of lower quality (lower star rating). Although the topic is important and policy relevant, I do not think it contributes sufficient new knowledge to the literature. There has been ample evidence showing black patients are more likely to bypass nearby high-quality hospitals and receive care in poorer quality hospitals, see e.g., D[i]mick et al. (2013).*

We acknowledge that the Dimick et al. (2013) is a pioneering paper on this subject, and we have (belatedly) cited their findings. However, as we explain in the introduction and the discussion, we believe that our result is more general because we consider all admissions rather than surgery (which, because it is usually scheduled in advance, provides more latitude in hospital choice); as well we consider urgent admissions where patient choice is less likely to play a role. Furthermore, we have demonstrated that this type of sorting (and lower levels of quality) are not uniform across markets and hospitals; as we show in this revision (and thanks to the excellent suggestions by reviewers), patient sorting is not uniform across the country, but is more prevalent in larger markets (as also shown in our previous paper), and the likelihood of being admitted to a lower quality hospital is lowest in hospitals exhibiting the lowest LHS (e.g., those hospitals in segmented markets for whom patients who are not Black are more likely to be admitted).

The following text has been added to the introduction:

While previous research has documented substantial racial disparities in hospital quality, often attributing differences to residential segregation, geography, or entrenched referral patterns (1-4), the mechanisms by which local patterns of hospital admissions contribute to these disparities have not been fully explored. Notably, prior studies, such as Dimick et al., examined how residential segregation and proximity influence Black patients’ likelihood of receiving care at low-quality hospitals(10). However, these approaches do not capture the systematic sorting of Black patients into lower-quality hospitals across the full range of admissions and across all available options in their immediate markets. This study extends the literature by introducing the Local Hospital Segregation (LHS) index, a novel measure that quantifies how representative each hospital’s patient population is relative to the racial composition of the surrounding market area. By applying this index to a national Medicare population and linking it to comprehensive, multidimensional hospital quality metrics, we offer new evidence of how within-market sorting beyond neighborhood residence drives inequities in the quality of care received by Black beneficiaries.

And later in the discussion:

The current study makes several significant contributions beyond prior work in this field. First, by employing the LHS index, we provide a direct and scalable approach to assess differential patterns of hospital admission within local markets, distinct from traditional measures based solely on residential segregation or broad regional patterns. Second, our analysis demonstrates that Black patients are more likely to be admitted to lower-quality hospitals even when higher-quality options are geographically proximate and network access is nominally equal—implicating within-market institutional and structural factors that perpetuate care disparities. Third, by leveraging comprehensive hospital quality ratings, our findings reveal that local hospital segregation is not linked to mortality, but is related to the remaining multiple domains of patient safety, experience, efficiency, and timeliness. Together, these findings shift the focus from geographic and patient-side explanations alone toward the recognition of supply-side and systemic barriers rooted in how hospitals admit and serve patients within their markets. This broader analysis thus may help to design policy interventions aimed at advancing equity in U.S. health care delivery.

7. *It would be more interesting if the authors could explore the potential mechanisms. For example, primary care physicians are patients’ entry points to the healthcare system. Is the reason black patients bypass high-quality hospitals because primary care physicians serving black communities tend to refer patients to specialists affiliated with poor-quality hospitals? If so, why?*

An excellent suggestion; we note that the Dimick et al. (2013) study considered a similar question, and referenced Bach et al. (2004) (which we now also cite); more recently Landon et al. (2021) and Popescu et al (2024) document large differences in access to physician specialists by racial group. Our text in the discussion now reads:

…the literature and our own results indicate that structural, economic, and institutional factors—such as insurance networks, hospital referral patterns, historical relationships, and experiences of inclusion or exclusion within health care settings—play a predominant role in shaping hospital selection for Black patients(8, 9, 29).

8. *The authors need to explain how they constructed the patient flow measure using Medicare claims data. Is it only based on the inpatient data, or both inpatient and outpatient data? What is the methodology? If we are only looking at hospital admissions based on inpatient claims, I would think the “hospital choice” is more determined by the referring physicians’ affiliation than the patients’ preferences. Can the authors explore outpatient choices as well?*

We apologize for the confusion and have rewritten our explanation for how we constructed the patient flow measure:

The measure captures the difference between the fraction of inpatient Black admissions at a given hospital and the corresponding fraction of Black admissions in its market area, defined as ZIP codes within a 30-minute drive of the hospital.

In the limitation section we also note that we do not calculate outpatient patient flows.

We acknowledge that our patient assignment is limited to inpatient hospital admissions, and that the patterns of outpatient visits in hospitals (or associated medical practices) may differ.

Unfortunately, our Data Use Agreement (DUA) with CMS precludes us from analyzing the outpatient data, which would be far more complex, particularly when patients seek outpatient care from multiple health care providers, some of whom might be connected with one hospital and others with different hospitals.

9. *Also, it is unclear if hospitals with high LHS are indeed of lower quality, or it is simply because CMS hospital star rating scores (not just the mortality scores) inadequately account for social risk factors. The paper mentions that their empirical findings reject the explanation of inadequate risk adjustment based on the insignificant association between mortality score and LHS, but this argument applies to the other group scores as well (e.g., readmission and safety).*

We acknowledge that we cannot rule out the possibility of bias in the hospital star rating scores, but the reason why we were reassured by the lack of association between the mortality score and the LHS is that we would have expected the mortality score to exhibit equal (or perhaps even greater) bias than the readmission and safety measures. That said, we now include a further limitation:

A final limitation is the possibility of bias in the hospital star rating scores because of inadequate risk adjustment. While the lack of association between the LHS and the mortality score does not point towards bias in the measurement of mortality, we note that other measures, such as readmission rates or safety, may exhibit a larger degree of bias.
